# Changes in the faecal bile acid profile in dogs fed dry food vs high content of beef: a pilot study

**DOI:** 10.1186/s13028-018-0383-7

**Published:** 2018-05-11

**Authors:** Kristin Marie Valand Herstad, Helene Thorsen Rønning, Anne Marie Bakke, Lars Moe, Ellen Skancke

**Affiliations:** 1 Department of Companion Animal Clinical Sciences, Faculty of Veterinary Medicine, Norwegian University of Life Sciences (NMBU), Oslo, Norway; 2 Matriks AS, Forskningsparken, Gaustadallèen 21, 0349 Oslo, Norway; 3 Department of Basic Sciences and Aquatic Medicine, Faculty of Veterinary Medicine, Norwegian University of Life Sciences (NBMU), Oslo, Norway

**Keywords:** Commercial dry food, Healthy client-owned dogs, Minced beef, Primary and secondary bile acids

## Abstract

**Background:**

Dogs are fed various diets, which also include components of animal origin. In humans, a high-fat/low-fibre diet is associated with higher faecal levels of bile acids, which can influence intestinal health. It is unknown how an animal-based diet high in fat and low in fibre influences the faecal bile acid levels and intestinal health in dogs. This study investigated the effects of high intake of minced beef on the faecal bile acid profile in healthy, adult, client-owned dogs (n = 8) in a 7-week trial. Dogs were initially adapted to the same commercial dry food. Thereafter, incremental substitution of the dry food by boiled minced beef over 3 weeks resulted in a diet in which 75% of each dog’s total energy requirement was provided as minced beef during week 5. Dogs were subsequently reintroduced to the dry food for the last 2 weeks of the study. The total taurine and glycine-conjugated bile acids, the primary bile acids chenodeoxycholic acid and cholic acid, and the secondary bile acids lithocholic acid, deoxycholic acid (DCA) and ursodeoxycholic acid (UDCA) were analysed, using liquid chromatography–tandem mass spectrometry.

**Results:**

The faecal quantities of DCA were significantly higher in dogs fed the high minced beef diet. These levels reversed when dogs were reintroduced to the dry food diet. The faecal levels of UDCA and taurine-conjugated bile acids had also increased in response to the beef diet, but this was only significant when compared to the last dry food period.

**Conclusions:**

These results suggest that an animal-based diet with high-fat/low-fibre content can influence the faecal bile acids levels. The consequences of this for canine colonic health will require further investigation.

**Electronic supplementary material:**

The online version of this article (10.1186/s13028-018-0383-7) contains supplementary material, which is available to authorized users.

## Background

Bile acids (BA) are essential for digestion and absorption of dietary lipids and lipid-soluble vitamins in the small intestine in mammals as well as in other vertebrates [1]. Studies mainly performed in cell-lines from humans and laboratory animals describe that BA also function as signalling molecules by activating receptors in the gall bladder, intestine and accessory digestive organs. These receptors and their ligands are involved in the regulation of lipid and glucose homeostasis [2–4] and they are believed to modulate the immune response in the liver and intestine [5]. However, high levels of some of these BA are toxic for colonic cells [6–8], and their concentrations are therefore tightly regulated [9].

The primary BA, cholic acid (CA) and chenodeoxycholic acid (CDCA) are synthetized from cholesterol and conjugate with either glycine or taurine in the liver. The latter is the most common in dogs [10, 11]. Most conjugated BA (> 95%) are reabsorbed in the ileum [12] and are returned to the liver through the enterohepatic circulation. BA that escape absorption, are deconjugated and converted through 7 alpha-dehydroxylation to secondary BA by colonic bacteria. The secondary BA deoxycholic acid (DCA) and lithocholic acid (LCA) originate from CA and CDCA, respectively [13]. Ursodeoxycholic acid (UDCA) is also produced by bacterial transformation from the primary BA CDCA [14].

Although dogs have adapted to a diet containing considerable amounts of carbohydrates through the domestication process, they were originally carnivores [15, 16]. In humans, a diet consisting of high content of animal derived protein and fat, and low content of carbohydrates, has been associated with increased faecal levels of BA, including DCA [8]. High levels of DCA may contribute to the formation and/or progression of colorectal tumours in humans [17] and mice [7, 18]. In contrast, UDCA is considered to have chemopreventative properties, and may counteract the effect of DCA, as demonstrated in human colon cancer cell lines [19, 20]. Colorectal tumours are rarely diagnosed in dogs [21, 22], yet they are considered more common in dogs than in other animal species [23]. Since similar molecular mechanisms have been described in the colorectal tumorigenesis in humans and dogs [24–26], and as dogs live in similar environments as humans, knowledge regarding how diet influences the faecal BA composition may be valuable for both dogs and humans.

Characterization of the pre- and postprandial serum concentrations of total BA aids in identifying impaired hepatic function and is useful in diagnosing portosystemic shunts (PSS) in dogs [27]. However, the various BA are rarely measured in faeces, and studies characterizing the canine faecal BA profile are sparse [28–30]. Furthermore, little is known about how a meat-based diet influences the levels of these BA.

The aim of this study was therefore to use liquid chromatography–tandem mass spectrometry (LC–MS/MS) to characterize the faecal BA profiles in healthy dogs before, during and after a diet with high content of boiled minced beef (MB).

## Methods

The study protocol was reviewed and approved according to the guidelines of the ethics committee at the Faculty of Veterinary Medicine and Biosciences, Norwegian University of Life Sciences (NMBU) (Approval Number: 14/04723-23). All dog-owners gave a written informed consent before participation and were informed that they could leave the study at any time.

### Animals, study design and diets

The study population consisted of a heterogeneous population of healthy client owned dogs (n = 11) of both gender and of various breeds and ages. They were included in a 7-week prospective dietary intervention study (Table 1). Three dogs did not complete the study due to loose faeces/diarrhoea (faecal score > 4.5, based on a five-point scale where grade 1 represents hard, dry faeces and grade 5 represents watery diarrhoea) [31]. Thus, eight dogs completed all the diet periods and were included in the present investigation. A detailed description of the study, the dogs and the diets have been described previously [32]. In brief, all the dogs were adapted to a commercial dry food diet for 2 weeks (CD1). Thereafter, each dogs received a mixture of boiled minced beef (MB) and CD diet for 3 weeks, where the MB was gradually increased in weekly increments at the expense of the CD diet. Water was added to the minced beef at a ratio of 3 parts MB:1 part water and simmered for 15 min or until the meat was completely cooked. The meat with any remaining water was mixed with the CD, cooled, and served. The amount of MB given each week was calculated to provide 25 (low minced beef, LMB), 50 (moderate minced beef, MMB) and 75 (high minced beef, HMB) percent of the dog’s total energy requirement. Finally, all the dogs were reintroduced to the original CD diet in the last 2 weeks of the study (CD2). The energy requirement for each adult dog was estimated according to information provided by the owner concerning type and amount of diet fed prior to the study and/or the range of 350–500 kJ ME × BW^0.75^ based on activity level, coat quality, body weight and body condition score [33]. The energy content in diets were kept constant for each dog throughout the study period. The calculated content of macronutrients for these diets were as follows: CD: 27.1/100 g dry matter (DM) proteins, 16.3/100 g DM lipids, 48.3/100 g DM nitrogen-free extract (NFE; carbohydrate-containing fraction) and 10.4/100 g DM fibre (non-starch polysaccharides); and HMB: 46.2/100 g DM proteins, 33.1/100 g DM lipids, 15.6/100 g DM NFE, and 3.4/100 g DM fibre. The detailed composition of the diets are found in Additional file 1.

**Table 1:** Demographic overview of the eight client-owned dogs included in a 7-week dietary intervention study

Dog no.^a^	Breed	Gender Female F/male M	Age (years)	Body weight (kg)
1	English Springer Spaniel	F	8	19.5
3	Small Munsterlander	F	6	21.5
4	Eurasier	F	1.5	17.7
5	Irish Setter	M	4	21.5
6	Mixed breed	M	5	14.7
7	English Setter	M	5	28
10	English Cocker Spaniel	F	8	10.3
11	German Shorthaired Pointer	F	3	19.9

^a^Dog no. 2, 8 and 9 did not complete all the diet periods

The data presented herein are from faecal samples collected and analysed from each of the dogs during the last 3 days from diet periods CD1 and HMB, and from the last 2 days from diet period CD2. All faecal samples analysed had normal faecal consistency. Samples were freeze-dried (Christ Alpha 1–4; SciQuip, Shropshire, UK) [34] and subsequently frozen and stored at − 80 °C prior to further processing.

### Sample preparation

Liquid chromatography–tandem mass spectrometry (LC–MS/MS) was used to analyse faecal BA. These included CA, CDCA, DCA, LCA, UDCA, and glycine- and taurine conjugated forms of these BA. A detailed overview of the BA are found in Additional file 2. The method for extraction of BA was based on Hagio et al. [35] with the following modifications: A total of 100 µL of 0.1 µg/mL internal standard was added to each freeze-dried faecal sample of 100 mg. Centrifugation of samples were performed at 4 °C. The evaporation steps were performed at room temperature. The methanol extracts were purified with solid phase extraction using an Oasis HLB cartridge (Waters, Milford, MA, USA), following the generic Oasis HLB protocol. The eluates were evaporated to dryness at room temperature under a stream of air and the dry residues were reconstituted in 1 mL methanol/10 mM ammonium acetate (1 + 1). The extracts were filtered through 0.22 µm nylon spin filters (Spin-X, Costar, Corning Inc., Corning, NY, USA) for 3 min at 11,000×*g*. The filtered extracts were transferred to HPLC-vials and subsequently stored at − 20 °C until LC–MS/MS analysis.

### Liquid chromatography–tandem mass spectrometry (LC–MS/MS)

The analysis was performed with an Agilent 1290 liquid chromatography system (Agilent Technologies, Waldbronn, Germany) coupled online with an Agilent G6490 triple quadrupole mass spectrometer (Agilent Technologies, Singapore) with a JetStream ESI ion source. The LC–MS/MS method described by Hagio et al. [35] was modified. The separation was done on a Waters Acquity BEH C18 column, 100 mm × 2.1 mm i.d. and 1.7 µm particles, with 10 mM ammonium acetate in water as mobile phase A and acetonitrile as mobile phase B (MPB). The flow rate was 0.4 mL/min and the column temperature 40 °C. The gradient started with 1 min 20% MBP, then went from 20 to 50% MPB in 9 min, then from 50 to 95% MBP in 0.1 min followed by 3 min in 95% MBP. The column was equilibrated in 20% MPB for 3 min before the next injection. Total analysis time was 15 min. The injection volume was 1 µL and the auto sampler temperature 4 °C.

All BA were ionized in negative mode and detected as their (M-H)—ions. The monitored ion transitions and compound specific parameters are given in Additional file 3a. All common MS/MS-parameters are provided in Additional file 3b.

Due to the ubiquitous presence of BA in faeces it was impossible to obtain a truly negative sample material. The method validation was therefore performed by spiking a pooled faecal sample with BA and subtracting the BA levels in the same sample without addition, to evaluate both linearity, precision and limit of detection. The precision study was done by spiking six samples at 100 µg/g. The linearity was evaluated from spiked samples at five levels; 0.1, 0.5, 1, 10 and 50 µg/g. Grade 1 water was used as negative control. The faecal BA concentrations were calculated relative to the spiked samples used to evaluate the precision. Therefore, this method is only semi-quantitative. The faecal BA concentrations are expressed in µg/g DM.

The precision at 100 µg/g was < 13% for all compounds. The limits of detection for all BA was 1 µg/g. Chromatograms of faecal BA from one dog (id 7), are shown in Additional file 4.

### Statistical methods

Data were tested for normality using the Shapiro–Wilk normality. Non-parametric Wilcoxon signed-rank test was used to calculate statistical differences between the various BA between the diet periods (CD1 vs HMB and CD2 vs HMB) without correction for multiple comparison. The software Graph Pad, PRISM v.7 (CA, USA) was used. A two-dimensional Principal component analysis (PCA) plot was generated using PRIMER7 [36]. A P value below 0.05 was considered statistically significant.

## Results

**Fig. 1:**
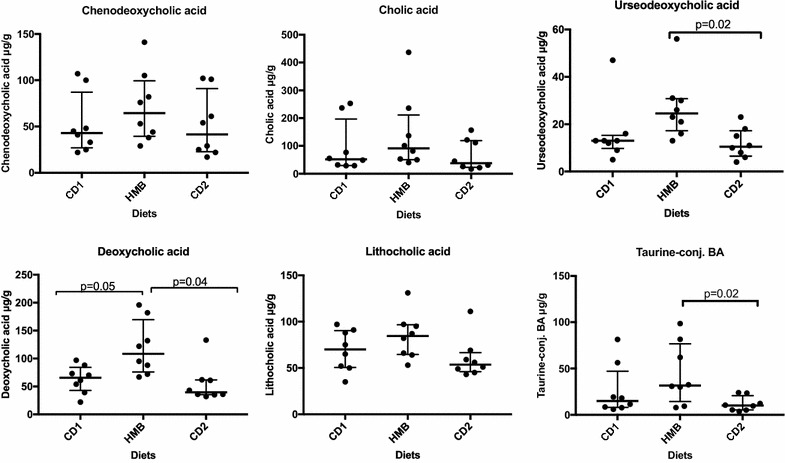
Median concentrations with interquartile ranges of bile acids (BA) (µg/g faeces) in samples of eight dogs fed commercial dry food at the start and end of the study (CD1 and CD2) and high minced beef (HMB). Significant differences of faecal BA in diet periods CD1 vs HMB and CD2 vs HMB are indicated (Wilcoxon signed-rank test without correction for multiple comparison). *CD1* Commercial dry food given the first 2 weeks of the study, *CD2* commercial dry food given the last 2 weeks of the study, *HMB* high minced beef, *CA* cholic acid, *CDCA* chenodeoxycholic acid, *DCA* deoxycholic acid, *LCA* litocholic, *UDCA* ursodeoxycholic acid, Taurine-conj. BA (taurine-conjugated CA, CDCA, DCA, and LCA)

The secondary BA, DCA were significantly higher in the HMB samples compared with the levels in both CD1 and CD2 samples (P = 0.05 and 0.04, respectively). Higher quantities of UDCA were detected in the HMB samples compared with that of CD2 samples (P = 0.02), but this was not significant when compared to CD1 samples (P > 0.1, Fig. 1). Although the median values for the primary BA, CA and CDCA were higher in HMB samples, the differences were not statistically significant (P > 0.1, Fig. 1). However, the levels of taurine-conjugated BA were significantly higher in the HMB samples compared with the CD2 samples (P = 0.02), but not compared with CD1 samples (P > 0.5). Concentrations of glycine-conjugated BA were measured, but were below quantification limit in all dogs (Table 2).

**Table 2:** Concentrations of faecal bile acids (µg/g)

Dog_id^a^	Diet	CA	CDCA	DCA	LCA	UDCA	G-DCA	G-LCA	T-CA	T-CDCA	T-DCA	T-LCA
1	CD1	32	41	54	52	13	1	1	3	1	1	0
	HMB	40	53	67	53	21	0	2	5	1	1	0
	CD2	112	61	36	43	8	4	2	2	1	1	0
3	CD1	55	45	73	65	16	2	1	5	2	2	0
	HMB	437	105	182	95	56	4	1	28	1	52	1
	CD2	122	102	62	59	23	0	2	4	1	1	0
4	CD1	49	48	97	97	13	5	1	19	7	41	14
	HMB	50	29	72	66	13	0	1	31	4	22	5
	CD2	26	25	43	56	11	0	1	11	2	7	4
5	CD1	29	25	61	75	12	2	1	5	2	8	3
	HMB	53	38	95	82	26	4	2	7	1	17	5
	CD2	22	22	36	49	10	2	1	1	0	2	1
6	CD1	29	22	39	50	9	2	1	2	1	5	3
	HMB	137	76	132	97	30	7	3	10	1	16	4
	CD2	17	17	32	45	6	6	2	2	1	5	2
7	CD1	77	33	22	35	5	8	1	2	1	3	2
	HMB	236	82	196	131	31	10	3	21	3	66	9
	CD2	31	29	35	51	4	5	2	3	1	4	2
10	CD1	253	107	88	88	13	8	2	8	2	7	2
	HMB	82	44	88	64	16	11	3	2	1	5	1
	CD2	157	101	133	111	18	18	3	7	2	11	4
11	CD1	237	100	70	91	47	0	1	29	7	15	6
	HMB	101	141	122	87	23	3	2	11	1	17	3
	CD2	45	54	61	69	15	5	2	3	1	6	2

The concentrations were determined semiquantitatively

*CA* cholic acid, *CDCA* chenodeoxycholic acid, *DCA* deoxycholic acid, *LCA* litocholic, *UDCA* ursodeoxycholic acid, glycine-conjugated DCA (G-DCA) and LCA (G-LCA), taurine-conjugated CA (T-CA), CDCA (T-CDCA), DCA (T-DCA), and LCA (T-LCA))

^a^Detailed demographics of these dogs are given in Table 1

**Fig. 2:**
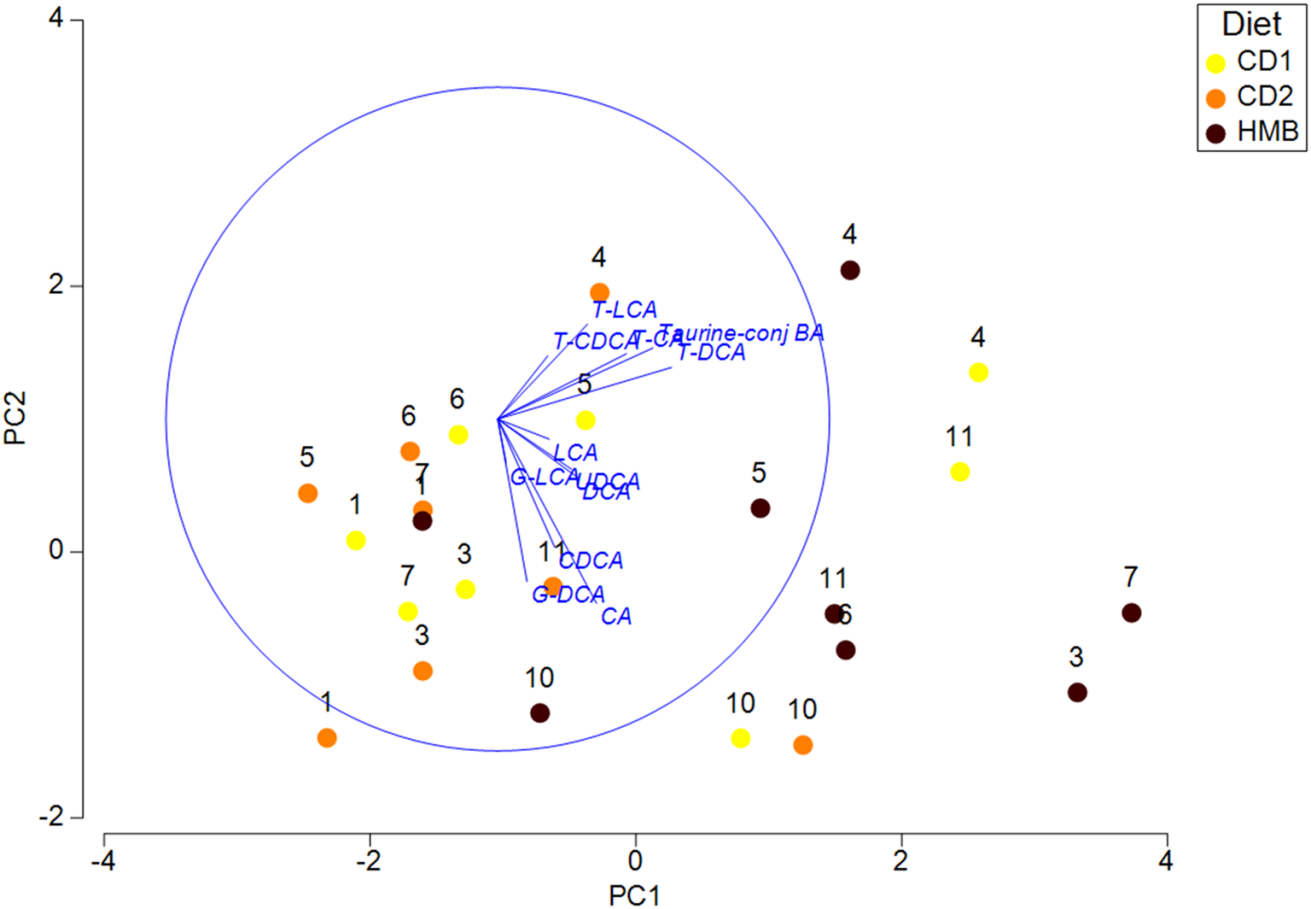
A Principal component analysis (PCA) plot showing the relationship between samples. The data are displayed across the two main principal components (PC1 and PC2). Each point represents one sample and each colour represents diet period. Closer clustering between points indicate higher relative commonality with respect to bile acid composition in those samples. Concomitantly, larger distances between points indicate lower relative commonality of bile acid composition in those samples. The first axis, PC1 accounted for 55% of the variability and PC2 accounted for 20% of the variability. The directions of the vectors (blue lines) corresponding to BA, particularly LCA, UDCA and DCA are directed towards the HMB samples. *CD1* Commercial dry food given the first 2 weeks of the study, yellow points; *CD2* commercial dry food given the last 2 weeks of the study, orange points, *HMB* high minced beef, black points, *CA* cholic acid, *CDCA* chenodeoxycholic acid, *DCA* deoxycholic acid, *LCA* litocholic, *UDCA* ursodeoxycholic acid, Taurine-conj. BA (taurine-conjugated CA, CDCA, DCA, and LCA)

As evaluated by a PCA plot, the majority of HMB samples are displayed along the first axis (PC1) and the vectors (bile acids), particularly LCA, DCA and UDCA, are directed towards the HMB samples (Fig. 2).

The variability in breed, age and body size between both genders of dogs made it impossible to perform any statistical testing for any possible impact of these factors on the faecal BA composition.

## Discussion

A diet shift from commercial dry food (CD) to high minced beef (HMB) and vice versa, during a 7-week dietary intervention study influenced faecal BA profiles in healthy client-owned dogs. Specifically, the secondary BA, DCA and UDCA increased in the HMB samples compared with the CD1 and/or CD2 samples, likely due to the presence of colonic bacteria with 7 alpha-dehydroxylating capabilities that transform primary BA to secondary BA. It is known that members within *Clostridium* and *Eubacterium* have this capability [13, 37]. We have previously reported, using the same study population, significantly higher relative abundances of an OTU in the family *Clostridiaceae* in the HMB samples [32]. This bacterial taxa was classified within a BLAST search to be *Clostridia hiranonis* with 97% identity. Interestingly, this species is capable of converting CA and CDCA into DCA and LCA, respectively [38]. Thus, the increased presence of this taxa may explain the higher faecal quantity of DCA in dogs fed HMB. The concomitant rise in the quantity of UDCA, rather than LCA, may indicate the possibility that increased bacterial transformation of CDCA to UDCA [14] is more likely to occur than bacterial transformation of CDCA to LCA in dogs. Moreover, the bacterial 7 beta-dehydroxylation of UDCA yield LCA [13, 39], but the low quantity of LCA may suggest that this process is not dominant in the intestine of dogs. However, since we used a semi-quantitative approach, these results needs to be validated in studies where the exact faecal quantities of BA are measured.

The apparent lack of glycine-conjugated BA in the faeces, yet detectable levels of taurine-conjugated BA, confirm that dogs primarily conjugate their bile acids with taurine rather than glycine [40–42]. Furthermore, the significantly higher taurine-conjugated BA levels measured in the faeces collected during the HMB period compared to the CD2 period suggest that the high lipid levels of the HMB diet can induce greater primary BA secretion. However, observed levels of primary BA, CA and CDCA were variable between dogs and not significantly increased in response to the HMB diet. The variable response between dogs in this study may be explained by differing BA metabolism, intestinal peristalsis, intestinal pH and/or gastrointestinal absorption of BA, as well as differences in the intestinal microbiota composition, which may result in different levels of secondary bile acid in response to diet in these individuals [1, 43].

The hydrophobicity of the BA influences their cytotoxic potential, ranking UDCA as the most hydrophilic and LCA as the most hydrophobic (BA hydrophobicity scale: UDCA < CA < CDCA < DCA < LCA) [44]. DCA has been shown to induce oxidative damage of DNA in vitro, which may result in abnormal cell proliferation of mutagenic, apoptosis-resistant cells [17, 45–47]. In contrast to the possible cytotoxic effects of DCA and LCA on colonic cells, UDCA is believed to have chemoprotective potential [19, 48]. A previous study of ten laboratory dogs described that oral treatment with UDCA resulted in lower ratio of secondary to primary BA [10]. Interestingly, the quantity of faecal UDCA in humans appear to be low in general [49], in contrast to the levels in dogs observed in this study. Whether dogs generally are adapted to having an intestinal microbiota that transform higher quantities of primary BA to UDCA compared to humans, also in response to a high-fat intake, merits further investigations.

In contrast to dietary fat, plant-fibre is thought to protect against colorectal cancer development in humans. Dietary fibres are fermented to short chain fatty acids (SCFA), which purportedly have anti-inflammatory and anti-carcinogenic properties [50]. One mode of action suggested is that the production of SCFA by bacterial fermentation of non-digestible carbohydrates reduces luminal pH and bacterial 7 alpha-dehydroxylase activity, and hence conversion of primary to the secondary BA, DCA and LCA is inhibited [51]. Fibres also bind to BA and thus facilitate their excretion [52]. Moreover, antioxidants in plants, such as beta-carotene and alpha-tocopherol may inhibit the detrimental effects of DCA on colonic cells [47]. In dogs, animal-fibres, such as collagen, has been suggested to have the same properties as plant-fibre [53], and thereby limit any potential toxic effects from secondary BA.

In humans, a diet with high content of protein and fat and low content of fibre, is associated with a higher risk of colorectal cancer [8, 54, 55]. Moreover, elevated serum and faecal levels of DCA have been observed in humans with colorectal adenoma and carcinoma compared with healthy controls [56, 57]. Dogs are fed various diets, which also include more animal-based diets preferred by some pet owners [58, 59]. Yet dogs rarely develop colorectal cancer [21, 60]. Given dogs’ carnivorous origins, it may not be surprising to find metabolic differences between humans and dogs that can explain differences in the risks of developing chronic intestinal, associated digestive organ and systemic diseases. For instance, dogs’ lipoprotein transportation of fat differs from that of humans [61], which may be the reason why atherosclerosis is not a major issue in dogs. Future studies should evaluate the faecal levels of BA, and particularly DCA and UDCA in dogs with colorectal cancer, non-tumour related colonic diseases, as well as healthy controls to gain an understanding of BA involvement in intestinal health in dogs.

The main limitation of this study was the small and heterogeneous sample size. Factors such as age, breed, body size/weight, gender, as well as previously fed diets may have influenced the faecal bile acid composition in our dogs. Previous studies have found that these aforementioned factors may influence the intestinal microbiota composition [62–65]. Whether the metabolites produced by the microbiota, including bile acids, also are influenced by these factors needs to be determined in future, adequately powered studies. Moreover, the influence of the individual dietary components, such as fat, starch, proteins, micronutrients, fibre, collagen etc., on the outcome was not tested. Although the discussion primarily focused on the influence of dietary fat, the presence and/or absence of other diet components most likely also influenced the faecal bile acid composition.

## Conclusions

A diet shift from commercial dry food to one of high beef content and vice versa, resulted in changes in the faecal BA profiles of healthy client-owned dogs. A high-fat/low-fibre diet in humans results in accumulation of secondary BA in the colon, particularly DCA, which has cytotoxic effects on colonic cells. Interestingly, our results in dogs revealed that the increase in DCA was accompanied by an increase in UDCA, the latter believed to have a chemoprotective mode of action. Since dogs have evolved from carnivorous wolves, and therefore presumed tolerant of high protein, high fat diets, they may have a different metabolism of BA, or have protective mechanisms against potential harmful effects induced by secondary BA, in order to maintain colonic health. Further studies are needed to more specifically evaluate the role of BA in colonic diseases of dogs.

## Additional files

**Additional file 1.** Ingredients and nutrient composition of the rations during the seven-week dietary intervention study (a), and main ingredients in CD (b).

**Additional file 2.** A detailed overview of the BA characterized by LC-MS/MS.

**Additional file 3.** The monitored ion transitions and compound specific parameters (a). Common MS/MS-parameters for all ion transitions (b).

**Additional file 4.** Chromatograms of faecal LCA (a), CA (b), DCA (c), CDCA (d) and UDCA (e) from one dog (id 7) fed commercial dry food the first two weeks of the study (CD1) and the last two weeks of the study (CD2) and high minced beef (HMB).

## Abbreviations

BA: bile acids
CA: cholic acid
CD: commercial dry food
CDCA: chenodeoxycholic acid
DCA: deoxycholic acid
DM: dry matter
HMB: high minced beef
LC–MS/MS: liquid chromatography–tandem mass spectrometry
LMB: low minced beef
MB: minced beef
MMB: moderate minced beef
MPB: mobile phase B
NFE: nitrogen-free extract
OTU: operational taxonomic unit
PCA: principal component analysis
SCFA: short chain fatty acid
UDCA: ursodeoxycholic acid
