# Supplementary material for: Changes in the faecal bile acid profile in dogs fed dry food vs high content of beef: a pilot study
Source: Acta Vet Scand. 2018 May 11;60:29. doi: 10.1186/s13028-018-0383-7 (PMC5948804; doi:10.1186/s13028-018-0383-7)
Supplement: Supplementary file 2 — Additional file 2. A detailed overview of the BA characterized by LC-MS/MS. [file 13028_2018_383_MOESM2_ESM.docx]

**Additional file 2. A detailed overview of the BA characterized by LC-MS/MS**

| Bile acid* | Chemical name | Chemical formula |
| --- | --- | --- |
| Cholic acid (CA) | 5b-cholanic acid-3a,7a,12a-triol | C_24_H_40_O_5_ |
| Chenodeoxycholic acid (CDCA) | 5b-cholanic acid-3a,7a-diol, | C_24_H_40_O_4_ |
| Deoxycholic acid (DCA) | 5b-cholanic acid-3a,12a-diol | C_24_H_40_O_4_ |
| Lithocohlic acid (LCA) | 5b-cholanic acid-3a-diol | [C_24_H_40_O_3_](https://pubchem.ncbi.nlm.nih.gov/search/#collection=compounds&query_type=mf&query=C24H40O3&sort=mw&sort_dir=asc) |
| Ursodeoxycholic acid (UDCA) | 5b-cholanic acid-3a,7b-diol | [C_24_H_40_O_4_](https://pubchem.ncbi.nlm.nih.gov/search/#collection=compounds&query_type=mf&query=C24H40O4&sort=mw&sort_dir=asc) |
| Taurocholic acid (T-CA) | 5b-cholanic acid-3a,7a,12a-triol-N-(2-sulpho-ethyl)-amide | [C_26_H_45_NO_7_S](https://pubchem.ncbi.nlm.nih.gov/search/#collection=compounds&query_type=mf&query=C26H45NO7S&sort=mw&sort_dir=asc) |
| Taurochenodeoxycholic acid (T-CDCA) | 5b-cholanic acid-3a,7a-diol-N-(2-sulpho-ethyl)-amide | [C_26_H_45_NO_6_S](https://pubchem.ncbi.nlm.nih.gov/search/#collection=compounds&query_type=mf&query=C26H45NO6S&sort=mw&sort_dir=asc) |
| Taurodeoxycholic acid (T-DCA) | [5b-cholanic acid-3a,12a-diol-N-(2-sulphoethyl)-amide] | [C_26_H_45_NO_6_S](https://pubchem.ncbi.nlm.nih.gov/search/#collection=compounds&query_type=mf&query=C26H45NO6S&sort=mw&sort_dir=asc) |
| Taurolithocohlic acid (T-LCA) | [5b-cholanic acid-3a-ol-N-(2-sulphoethyl)-amide] | [C_26_H_45_NO_5_S](https://pubchem.ncbi.nlm.nih.gov/search/#collection=compounds&query_type=mf&query=C26H45NO5S&sort=mw&sort_dir=asc) |
| Glycocholic acid (G-CA) | [5b-cholanic acid-3a,7a,12a-triol-N-(carboxymethyl)-amide] | [C_26_H_43_NO_6_](https://pubchem.ncbi.nlm.nih.gov/search/#collection=compounds&query_type=mf&query=C26H43NO6&sort=mw&sort_dir=asc) |
| Glycochenodeoxycholic acid (G-CDCA) | ([5b-cholanic acid-3a,7a-diol-N-(carboxymethyl)-amide | [C_26_H_43_NO_5_](https://pubchem.ncbi.nlm.nih.gov/search/#collection=compounds&query_type=mf&query=C26H43NO5&sort=mw&sort_dir=asc) |
| Glycodeoxycholic acid (G-DCA) | [5b-cholanicacid-3a,12a-diol-N-(carboxymethyl)-amide] | [C](https://pubchem.ncbi.nlm.nih.gov/search/#collection=compounds&query_type=mf&query=C26H43NO5&sort=mw&sort_dir=asc)_[26](https://pubchem.ncbi.nlm.nih.gov/search/#collection=compounds&query_type=mf&query=C26H43NO5&sort=mw&sort_dir=asc)_[H](https://pubchem.ncbi.nlm.nih.gov/search/#collection=compounds&query_type=mf&query=C26H43NO5&sort=mw&sort_dir=asc)_[43](https://pubchem.ncbi.nlm.nih.gov/search/#collection=compounds&query_type=mf&query=C26H43NO5&sort=mw&sort_dir=asc)_[NO](https://pubchem.ncbi.nlm.nih.gov/search/#collection=compounds&query_type=mf&query=C26H43NO5&sort=mw&sort_dir=asc)_[5](https://pubchem.ncbi.nlm.nih.gov/search/#collection=compounds&query_type=mf&query=C26H43NO5&sort=mw&sort_dir=asc)_ |
| Glycolithocohlic acid (G-LCA) | ([5b-cholanic acid-3a-ol-N-(carboxymethyl)-amide] | [C_26_H_43_NO_4_](https://pubchem.ncbi.nlm.nih.gov/search/#collection=compounds&query_type=mf&query=C26H43NO4&sort=mw&sort_dir=asc) |

| *Internal standard* |  |  |
| --- | --- | --- |
| Deoxycholic acid  98 atom % D, 98% (CP) | **7-Deoxycholic acid,-2,2,4,4-d_4_, 3a,12a-Dihydroxy-5ß-cholanic acid-2,2,4,4-d_4_** | 2,2,4,4-d_4_ |

*All bile acids were purchased from Sigma-Aldrich, St. Louis, Missouri, USA.
