# Supplementary material for: Changes in the faecal bile acid profile in dogs fed dry food vs high content of beef: a pilot study
Source: Acta Vet Scand. 2018 May 11;60:29. doi: 10.1186/s13028-018-0383-7 (PMC5948804; doi:10.1186/s13028-018-0383-7)
Supplement: Supplementary file 3 — Additional file 3. The monitored ion transitions and compound specific parameters (a). Common MS/MS-parameters for all ion transitions (b). [file 13028_2018_383_MOESM3_ESM.docx]

**Additional file 3a**. **The monitored ion transitions and compound specific parameters**

| Bile acid | Precursor ion | Product ion | Retention time (min) | Collision energy (eV) |
| --- | --- | --- | --- | --- |
| CA | 407.1 | 407.1  343.1 | 5.5 | 0  40 |
| CDCA | 391.1  391.1 | 391.1  345.1 | 7.3 | 0  40 |
| LCA | 375.1 | 375.1 | 10.0 | 0 |
| DCA | 391.1  391.1 | 345.1  391.1 | 7.5 | 40  0 |
| UDCA | 391.1  391.1 | 391.1  345.1 | 5.5 | 0  40 |
| G-CA | 464.3 | 464.3 | 8.7 | 0 |
| G-CDCA | 448.3 | 448.3 | 6.3 | 0 |
| G-LCA | 432.3 | 432.3 | 8.1 | 0 |
| G-DCA | 448.3 | 448.3 | 6.7 | 0 |
| G-UDCA | 448.3 | 448.3 | 4.9 | 0 |
| T-CA | 514.3 | 514.3 | 5.4 | 0 |
| T-CDCA | 498.3 | 498.3 | 6.7 | 0 |
| T-LCA | 482.3 | 482.3 | 8.5 | 0 |
| T-DCA | 498.3 | 498.3 | 7.0 | 0 |
| DCA-D4 | 395.1 | 395.1 | 7.5 | 0 |

**Additional file 3b: Common MS-parameters for all ion transitions**

| Fragmentor voltage (V) | 380 |
| --- | --- |
| Dwell time (ms) | 20 |
| CAV (V) | 5 |
| Gas temperature (°C) | 250 |
| Gas flow (L/min) | 13 |
| Nebulizer pressure (psi) | 40 |
| Sheath gas (°C) | 350 |
| Sheath gas flow (L/min) | 11 |
| Capillary (V) | 4500 |
| Nozzle (V) | 1000 |
| High pressure RF (iFunnel) | 150 |
| Low pressure RF (iFunnel) | 60 |
