# Supplementary material for: Changes in the faecal bile acid profile in dogs fed dry food vs high content of beef: a pilot study
Source: Acta Vet Scand. 2018 May 11;60:29. doi: 10.1186/s13028-018-0383-7 (PMC5948804; doi:10.1186/s13028-018-0383-7)
Supplement: Supplementary file 4 — Additional file 4. Chromatograms of faecal LCA (a), CA (b), DCA (c), CDCA (d) and UDCA (e) from one dog (id 7) fed commercial dry food the first two weeks of the study (CD1) and the last two weeks of the study (CD2) and high minced beef (HMB). [file 13028_2018_383_MOESM4_ESM.docx]

**Additional file 4. Chromatograms of faecal LCA (a), CA (b), DCA (c), CDCA (d) and UDCA (e) from one dog (id 7) fed commercial dry food the first two weeks of the study (CD1) and the last two weeks of the study (CD2) and high minced beef (HMB)**

HMB CDHHgfgHMBHMB

CD2 HHHgfgHMBHMB

CD1 HHgfgHMBHMB

LCA

4a: LCA (MRM transition 375.1 - 375.1) from the same dog fed CD1, CD2 and HMB.

CD1 CDHHgfgHMBHMB

CD2 CDHHgfgHMBHMB

HMB

CA

4b: CA (MRM transition 407.1 – 343.4) from the same dog fed CD1, CD2 and HMB.

HMBCDHHgfgHMBHMB

CD2 – CDHHgfgHMBHMB

CD1 – CDHHgfgHMBHMB

DCA

4c: DCA (MRM transition 391.1 – 345.1) from the same dog fed CD1, CD2 and HMB.

HMB CDHHgfgHMBHMB

CD2 – CDHHgfgHMBHMB

CD1 – CDHHgfgHMBHMB

CDCA

4d: CDCA (MRM transition 391.1 – 345.1) from the same dog fed CD1, CD2 and HMB.

HMB CDHHgfgHMBHMB

CD2 – CDHHgfgHMBHMB

CD1 – CDHHgfgHMBHMB

UDCA

4e: UDCA (MRM transition 391.1 – 345.1) from the same dog fed CD1, CD2 and HMB.
